# The Effect of Air Pollution Control Auditing on Reducing Carbon Emissions: Evidence from China

**DOI:** 10.3390/ijerph192417019

**Published:** 2022-12-18

**Authors:** Chen Zhao, Jiaxuan Zhu, Zhiyao Xu, Yixuan Wang, Bin Liu, Lu Yuan, Xiaowen Wang, Jiali Xiong, Yiming Zhao

**Affiliations:** 1 Institute of Natural Resources and Environmental Audits, School of Government Audit, Nanjing Audit University, Nanjing 211815, China; 2 School of Geographic and Biologic Information, Nanjing University of Posts and Telecommunications, Nanjing 210023, China

**Keywords:** air pollution control auditing, carbon emission, carbon emission intensity, mechanism

## Abstract

Analyzing the carbon-emission-reduction mechanism from the perspective of air pollution control auditing is of great practical significance for China to implement the dual-carbon strategy. Based on the panel data of 30 Chinese provinces from 2004 to 2018, we examine whether and how the auditing of air pollution control has an impact on carbon emission reduction by using multiple regression method and the mediating analysis. Our analyses show that air pollution control auditing can significantly restrain carbon emissions but has no impact on carbon emission intensity. Further research suggests that (1) the bottom-up audit represented by local audit institutions is more effective than the top-down audit represented by the National Audit Office; (2) air pollution control auditing follows a simple and direct method to curb carbon emissions by output reduction, regulation, and shutdown, rather than promoting technological progress and green transformation of enterprises in a high-quality development mode. Those findings provide an improvement direction for air pollution control auditing to contribute to carbon emission reduction and supply relevant policy references for implementing the dual carbon strategy.

## 1. Introduction

The global warming, mainly caused by excessive emissions of carbon dioxide, has had a disastrous impact on the world [1,2,3]. Global warming not only leads to temperature rises but also determines other indirect effects, such as ice melt at the poles and a decline in species diversity and extreme weather events. The threat of global warming to human beings is real, and the damage it does is incalculable [4,5]. It is a common goal for all countries and mankind to reduce carbon emissions and mitigate global warming.

Over the past 40 years of reform and opening up, China’s economy has developed at a high speed. However, along with significant achievements in economic construction, the massive use of fossil energy leads to the surge in carbon emissions. As the largest carbon emitter in the world, the task of carbon emissions in China is arduous. China has adopted many policies, measures, and actions to promote green development and set forth the carbon peak and carbon neutralization, i.e., the dual carbon goal by 2030 and 2060, as one of the essential national strategies [6,7].

As an important part of the national supervision mechanism, environmental auditing plays a critical role in reducing potential environmental pollution and promoting better environmental governance. Recently, China has successively implemented environmental audits on environmental policies, special funds, treatment projects, and leading cadres, including a series of special audits conducted by the audit institutions on the subject of air pollution control. Through air pollution control auditing, the audit institutions reveal the related problems in the implementation of the pollution control policy and the allocation and use of funds, restricting the non-compliance in the pollution control. Ultimately, they propose the measures to improve the regional pollution control mechanism.

Most of the current research on carbon emission reduction focuses on the relationship between carbon emissions and energy consumption structure [8,9], spatial industrial agglomeration [10,11], technological innovation [12,13], environmental regulation [14,15], and industrial structure upgrading, while the research on its impact on carbon emission reduction from the perspective of an audit supervisory mechanism is relatively rare, making it urgent to conduct research on it to provide empirical feedback for air pollution control auditing in carbon emission reduction and policy ideals for the country to realize dual carbon goal at an early date. The supervisory mechanism can directly or indirectly affect carbon emissions through resource allocation, policy orientation, institutional environment, and other factors. This study aims to clarify whether and how the air pollution control auditing can help promote carbon emission reduction. Compared with previous literature, the main contributions of this study include the following two aspects. On one hand, the theoretical channel of the influence of air pollution control auditing on carbon emissions is proposed and verified with the help of the regression and mediated-effects models, which improve the inherent logical relationship. On the other hand, the research into carbon emissions from the perspective of audit supervision is not only a relevant supplement to the existing studies, but also helps the government better understand and utilize the carbon emission government function of audit to better restrain the growth of carbon emissions.

The remainder of this paper is organized as follows. Section 2 reviews the relevant literature and proposes the Hypotheses. In Section 3, the variables, the data, and the model used are explained. Section 4 presents the results of the model estimations. Section 5 summarizes the main conclusions and provides the possible policy implications.

## 2. Literature Review and Research Hypotheses

### 2.1. The Influencing Factors of Carbon Emission

The influencing factors of carbon emissions are widely discussed. Most scholars believed that the energy consumption structure was an important leading and restrictive factor affecting regional carbon emissions [8,9]. Energy structure transformation and energy efficiency improvement were effective ways to reduce carbon emissions [16,17]. The relationship between industrial agglomeration and carbon emissions displayed an inverted U-shaped curve. When the industrial agglomeration reached a certain threshold, it could promote carbon emission reduction [10,11]. Wen and Liao [18] used a spatial econometric model to find that industrial agglomeration significantly increased the carbon emissions in the local region, while curbing the carbon emissions in the neighboring areas. The conclusions regarding the impact of technological innovation on carbon emissions were inconsistent. Some scholars claimed that technological innovation could effectively promote carbon emission reduction [12,19,20,21], while others believed that technological innovation had the opposite effect, resulting in increased carbon emissions [13,22,23,24]. Pao et al. [25,26] even proposed that technological process can initially increase carbon emissions and decrease them later on. The “green paradox” theory and the “forced emission reduction” theory were also formed about the effect of the environmental regulation on carbon emissions. Yin [27] confirmed that the direct effect of environmental regulation on carbon emissions showed a U-shaped curve, and environmental regulation had an indirectly impact on carbon emissions through the paths of energy consumption structure, industrial structure, technological innovation, and foreign direct investment. Ulucak et al. [14] confirmed the positive role of environmental regulation on carbon emission reduction, and the current environmental control measures were successful in achieving pollution reduction targets in Brazil, Russia, India, China, and South Africa.

### 2.2. Direct Impact of Air Pollution Control Auditing on Carbon Emissions

In terms of promoting carbon emission reduction by auditing, scholars mainly focused on energy auditing and environmental auditing. Energy auditing is a tool for diagnosing and analyzing energy consumption in the field of industry and construction and can reduce carbon emissions by improving energy efficiency. The research into the industry showed that it was an effective way to promote industrial energy efficiency by incorporating energy auditing into a broader energy use program [28,29]. Some empirical studies have confirmed that energy auditing could reduce energy consumption in households and small enterprises [30,31,32]. Energy auditing could overcome energy efficiency barriers that are the result of information asymmetry and accelerate the adoption of energy conservation measures by small business organizations, thus reducing energy consumption [33,34,35]. As an endogenous “immune system” in the national governance system, environmental auditing has the functions of prevention, disclosure, and defense. The existing studies showed that the emission reduction and energy conservation effect of environment auditing was obvious, which reflected the reduction of carbon emissions [35,36,37]. Zeng and Li [38] pointed out that environmental auditing mainly fulfilled an environmental governmental function by improving the implementation of environmental protection funds and promoting the implementation of environmental protection policies. As a part of environmental auditing, air pollution control auditing is based on the examination and verification of the government in the implementation of pollution abatement and energy-saving liabilities. It also helps to monitor the evaluative role in pollution control and energy conservation and promotes the carbon emission reduction.

### 2.3. Indirect Impact of Air Pollution Control Auditing on Carbon Emissions

The choice of local government behavior was a critical factor for carbon emissions, while auditing had a remarkable impact on local government [39]. The effect of local atmospheric environment treatment depended mainly on the industrial structure and the pollutant discharge status of enterprises [27,40,41]. On this basis, air pollution control auditing compelled local governments to take two strategies to curb carbon emissions. First, the local governments strove to strengthen the supervision of industrial enterprises and the punishment of enterprises with non-compliance [42]. Relying on their authority, local governments took simple and direct measures to reduce carbon emissions by limiting output, forcing power outage, and controlling the shutdown of heavy-polluting industrial enterprises. This approach worked quickly, but the “one size fits all” approach would lead to the reduction of local industrial output and industrial added value, thus hindering the local economic development. Second, local governments reduced carbon emissions by increasing the investment [42,43,44]. Anderson and Newell [45] found that government subsidies may be more effective at improving energy efficiency than other policy tools based on the analysis of government-sponsored energy auditing. Andersson et al. [46] believed that governmental industrial energy auditing programs subsidizing the companies to perform an energy auditing was an effective measure to help enterprises improve energy efficiency and further studied the evaluation criteria of energy auditing policies on this basis. Therefore, with the provision of external incentive measures such as financial subsidies, technological innovation and green transformation [47,48], the enterprise had the power to improve its energy efficiency and introduce advanced industrial waste gas treatment facilities, promoting the industrial development with low pollution, low energy consumption, and high added value, thus promoting carbon emission reduction. According to the above theoretical analysis, this paper draws the path map of the influence of air pollution control auditing on carbon emissions, and proposes Hypotheses 1, 2, and 3.

**Hypothesis 1.**  *Air pollution control auditing can significantly reduce carbon emissions and carbon emission intensity*.

**Hypothesis 2.**  *Air pollution control auditing reduces carbon emissions by shutting down high emission and high energy consumption enterprises*.

**Hypothesis 3.**  *Air pollution control auditing reduces carbon emissions by promoting the technological progress and green transformation of enterprises*.

## 3. Materials and Methods

### 3.1. Data

In this paper, the research samples selected consist of the panel data of 30 provinces in China (excluding Tibet, Hong Kong, Macao, and Taiwan) from 2004 to 2018. Our sample period ends in 2018, as the data related to air pollution control auditing end in 2018. The energy consumption data in the carbon emission calculation, the statistical data for control variables, and mediating variables are sourced from China Statistical Yearbook. The data about air pollution control auditing is collected from China audit Yearbook.

### 3.2. Variables

#### 3.2.1. Dependent Variable

Most studies on carbon emissions only consider carbon dioxide [41]. In this paper, there are two indicators for measuring carbon emissions. One is carbon emissions, which refers to the total amount of carbon dioxide emissions, the other is carbon emission intensity (i.e., carbon emissions per unit of GDP), which is a relative indicator. Both carbon emissions and carbon emission intensity are set as the dependent variable. The method of the United Nations Intergovernmental Panel on climate change (IPCC) is adopted to calculate the carbon emissions. The energy types considered are the 10 fossil energy sources, including coal, coke, crude oil, gasoline, kerosene, diesel, fuel oil, natural gas, liquefied petroleum gas, and electric power. The formulae are as follows

(1) $$C^{t}=\sum^{\;}i\sum^{\;}{j\;C}_{\mathrm{ij}}^{t}$$

(2) $$C_{\mathrm{ij}}^{t}=E_{\mathrm{ij}}^{t}\times r_{j}$$

(3) $$r_{j}=F_{j}\times e\times {\mathrm{EF}}_{j}\times O_{j}\times \frac{44}{12}$$

where $C^{t\;}$ denotes the total carbon dioxide emissions generated by each province, $C_{\mathrm{ij}}^{t}\;$ is the carbon dioxide emissions from the use of energy j in region I, $E_{\mathrm{ij}}^{t}$ represents 10 kinds of energy consumption, $r_{j\;}$ refers to the carbon emission coefficient corresponding to each energy source, $F_{j}\;$ represents the conversion coefficient of standard coal, e  is calorific value of standard coal, ${\mathrm{EF}}_{j}\;$ means the carbon content per unit calorific value of the energy, $O_{j\;}$ represents the carbon oxidation rate of the fuel, and $\frac{44}{12}\;$ indicates the conversion coefficient from carbon to carbon dioxide. The carbon emission coefficients of various energy sources are shown in Table 1.

#### 3.2.2. Independent Variable

The dummy variable audit is set as the independent variable to indicate whether the province has carried out air pollution control auditing. The government audit organizations in China are composed of the National Audit Office and local audit institutions. Previous studies suggested that the former has higher auditing independence and audit quality than the latter [40,49,50]. Thus, this paper further divides air pollution control auditing into the top-down audit and bottom-up audit. The top-down audit refers to the auditing of air pollution control implemented by the National Audit Office, and the bottom-up audit refers to those implemented by local audit institutions. When the National Audit Office or local auditing institution performs air pollution control auditing in the year, the value is set to be 1, otherwise it is set to 0.

#### 3.2.3. Mediating Variable

In order to analyze the influence mechanism of the air pollution control auditing on carbon emissions, mediating variables are needed to describe the carbon emission reduction supervision mode of governments [51,52]. An increase in industrial added value signifies a growth in economic scale [53]; thus, the industrial added value is selected as a mediate variable to measure the direct control. The mediate variable also chooses the numbers of industrial pollution control waste gas projects, the processing capacity of industrial waste gas treatment facilities, and the operating cost of industrial waste gas treatment facilities to measure the way to promote the green transformation of enterprises.

#### 3.2.4. Control Variable

In order to avoid other factors interfering with the results, six control variables are set to control the impact of the economic development, the industrial structure, the energy structure, and the pollution prevention and control. pgdp represents the per capita GDP; a higher pgdp will lead to more carbon emissions [54]. Ind represents the ratio of secondary industry value-added to GDP. When industry accounts for a large proportion of the economic structure, it is harder to reduce carbon emissions [55]. Energy represents the ratio of coal consumption to total energy consumption. Coal occupies a dominant position in China’s energy consumption structure; the higher proportion of coal consumption may result in more carbon emissions [56]. Tech represents the ratio of GDP to total energy consumption, i.e., the impacts on carbon emissions from technical level. Urban represents the ratio of urban population to total population. With the acceleration of urbanization, a large amount of energy will be consumed with the acceleration of urbanization, increasing carbon emissions [57]. Regulation represents the proportion of industrial-pollution-source treatment investment in GDP. Higher environmental regulation intensity will force enterprises to carry out technological innovation and choose clean energy to reduce carbon emissions [58]. The summary of variables is shown in Table 2.

### 3.3. Modeling

#### 3.3.1. Regression Analysis

To assess the influence of the air pollution control auditing on carbon emissions, a fixed effect model is established. The model is as follows

(4) $$\mathrm{Ln}\_{\mathrm{Carbon}}_{\mathrm{it}}=\beta_{0}+\beta_{1}{\mathrm{AA}}_{\mathrm{it}}+{\gamma Z}_{\mathrm{it}}+\mu_{i}+\delta_{i}+\varepsilon_{\mathrm{it}}$$

(5) $${\mathrm{Intensity}}_{\mathrm{it}}=\beta_{0}+\beta_{2}{\mathrm{AA}}_{\mathrm{it}}+{\gamma Z}_{\mathrm{it}}+\mu_{i}+\delta_{i}+\varepsilon_{\mathrm{it}}$$

where $\mathrm{Ln}\_{\mathrm{Carbon}}_{\mathrm{it}}\;$ and ${\mathrm{Intensity}}_{\mathrm{it}}$, respectively, represent the carbon emission and carbon emission intensity of province i in the year t; ${\mathrm{AA}}_{\mathrm{it}\;}$ indicates whether province i implements air pollution control auditing in year t; $Z_{\mathrm{it}\;}$ represents a series of control variables; $\mu_{i}\;$ represents the individual fixed effect of province i that does not change with time; and $\varepsilon_{\mathrm{it}\;}$ represents the random error term that this model cannot explain.

#### 3.3.2. Mediating Effect Analysis

In order to explore the possible indirect transmission mechanism of air pollution auditing on carbon emissions, the bootstrap method is adopted. Mackinnon et al. [59] suggested that the bootstrap method yields the most accurate confidence intervals for indirect effects. Compared with the stepwise regression method, the bootstrap method does not require the independent variable to have a significant effect on the dependent variable but directly tests the significance of the mediating effect coefficient, which can effectively avoid the influence of the “shading effect” on the results. Thus, the bootstrap method can be a good option to analyze an indirect effect [60,61,62]. The confidence intervals in this paper are all based on bootstrapping with 1000 iterations. If the bootstrapping confidence interval does not contain zero, the experimental factor is significant.

#### 3.3.3. Robustness Test

The robustness test of the benchmark regression is carried out, respectively, by four methods, including independent variable replacement, common trend test, propensity score matching, and a placebo test. In the first method, the intensity of the air pollution control auditing, which is defined as the frequency of implementing air pollution control auditing (AAF for short), is used to replace the original independent variable AA and then conduct the benchmark regression. The second method for the robustness test is the common trend test. Due to the different points in time that an air pollution control auditing was implemented in each province, we find that the numbers of provinces implementing air pollution control auditing before 2008 are significantly smaller than those after. Specifically, the average number is 1 before 2008 and 14 after 2008. The sample data are classified into treatment groups, which are defined as the provinces that have implemented air pollution control auditing for more than five years and control groups, which are defined as the remaining provinces. Therefore, the treatment group and control group should have a common developing trend before 2008.

Owing to the limitation of the cognitive level and available data, existing selection biases and some important variables for the empirical model being missing could lead to serious endogeneity. Considering the possible selection biases, the propensity score matching method is applied to test the endogeneity by taking the control variable as the covariate, and the nearest neighbor matching method is used in a 1:1 ratio to match. Then, a new benchmark regression is performed based on the new control variables and testing the potential endogeneity. We also control the endogeneity using a placebo test. Current research on the placebo test of panel data usually adopts regional randomization or regional–temporal randomization. The latter scheme is adopted for the placebo test in this paper, as the air pollution control auditing is conducted asynchronously.

## 4. Empirical Results and Analysis

### 4.1. Descriptive Statistics

The descriptive statistical results were summarized in Table 3. The maximum and minimum values of the carbon emissions are 11.91 and 7.13, respectively, and the standard deviation is 0.805, which reflects the large difference in carbon dioxide emissions in different regions. The minimum and maximum values of carbon emission intensity are 0.25 and 14.77, respectively, and the standard deviation is 2.418, which indicates that there is a gap in the economic benefits of carbon emissions in different regions. The average values of the top-down and bottom-up forms of air pollution control auditing are 0.218 and 0.162, respectively, indicating that the current implementation of air pollution control auditing is not strong enough.

### 4.2. Benchmark Regression Results

The Benchmark regression results are listed in Table 4. Column (1) and column (2), respectively, examine the impact of air pollution control auditing on carbon emissions and carbon emission intensity. The results in Column (1) show that the regression coefficient between air pollution control auditing and carbon emissions is significantly negative (−0.021), implying that air pollution control auditing has an inhibitory effect on the carbon emissions.

This conclusion is similar to some existing studies [37,63], however, which examine carbon emission reduction from the perspective of the function of government auditing in national governance. The carbon emission reduction influenced by air pollution control auditing is mainly attributed by the supervisory deterrence of audits, which could reveal problems such as the illegal use of the huge funds, long-term idle pollution-treatment facilities, ineffective implementation of pollution control projects, and other issues.

The regression coefficient between air pollution control auditing and carbon emission intensity in Column (2) is positive (0.021), which indicates that air pollution control auditing has no restrictive effect on carbon emission intensity. Furthermore, the influences of top-down audits on carbon emissions and carbon emission intensity are listed in Column (3) and Column (4), respectively. The estimated coefficients are positive, indicating that top-down audit has no inhibitory effect on carbon emissions and carbon emission intensity. The results of bottom-up audit are basically consistent with air pollution control auditing, as shown in Columns (5)–(6), which indicates that bottom-up audit significantly inhibits carbon emissions but has no impact on carbon emission intensity.

The results imply that bottom-up audit is more effective than top-down audit. On one hand, this may be due to the fact that the top-down supervision mode implements a “mandatory” audit supervision, leading to the audit work taking place in a passive situation, while the bottom-up audit supervision mode can provide full insight into the work initiative of auditors. On the other hand, the execution efficiency of local audit institutions is greater than that of the National Audit Office [64]. In addition, no matter what audit form is used, there is no significant inhibitory effect on carbon emission intensity. The reason for this is that carbon emission intensity is a relative indicator, which represents the ratio of carbon emissions to GDP. To complete the carbon emission reduction tasks issued by the state, some provinces may control their economic growth rates to reduce energy consumption and sacrifice the economy to reduce carbon emissions as necessary. Therefore, the benchmark regression results support Hypothesis 1, namely that air pollution auditing has an obviously impact on carbon emission reduction.

### 4.3. Mechanism of the Air Pollution Control Auditing

Hypotheses 2 and 3 are examined based on a mediating analysis to further analyze the working mechanism of air pollution control auditing to help reduce carbon emissions. The results are listed in Table 5. Firstly, the indirect effect of air pollution control auditing on carbon emission reduction by reducing industrial added value is −0.0099, which is significant at the 5% significant level. The corrected confidence interval is from −0.0168 to −0.0025, excluding zero, indicating that industrial added value plays a mediating role between the air pollution control auditing and carbon emissions. The intermediary effect accounts for 46.70% of the total effect. The bottom-up audit shows a similar result but has the indirect effect value of −0.0081. Secondly, the indirect effect of air pollution control auditing on carbon emissions by improving the treatment capacity of industrial waste gas treatment facilities is −0.0003, and the corrected confidence interval is from −0.0011 to 0.0005, containing zero, suggesting that improving the treatment capacity of industrial waste gas treatment facilities has no significant intermediary effect between air pollution control auditing and carbon emissions. Finally, corrected confidence intervals of implementing industrial pollution control waste gas projects and increasing the operating costs of industrial waste gas treatment facilities contain zero, which indicates that the air pollution control auditing cannot significantly curb carbon emissions by implementing relevant projects and increasing related investments.

Therefore, although the air pollution control auditing has the ability to directly reduce carbon emissions with its supervisory deterrence, the local government, when forced by air pollution control auditing, insists on direct control and reduction of output rather than promoting technological processes and green transformations, which is consistent with previous studies [65,66]. The results prove Hypothesis 2 but reject Hypothesis 3.

The principal reasons are concluded from the following two aspects in this paper. Firstly, the direct control has an immediate and perceptible effect to better meet the requirements of the local government’s performance assessment. For example, power rationing and discontinued production of the existing “two high” projects or enterprises such as coal-fired power plants and steel plants can effectively reduce carbon emission within a short time. Additionally, compared to technological innovation and green transformation, the direct control requires lower technological investment. Secondly, the supervisory role of air pollution control auditing in carbon emissions by local governments is not entirely fulfilled. In addition to carrying out investigations and providing suggestions, the audit institutions in China are also authorized to check on rectification results and force audited bodies to make sufficient rectifications. However, some audit institutions may only focus on the former, and the audit rectifications are ignored. This leads to the fact that audited bodies only make rectifications and corrections on the basis of specific problems instead of tackling them from the source.

### 4.4. Robustness Test

The benchmark regression results, calculated by replacing the independent variable AA with AAF, are shown in Columns (3)–(4) of Table 6. It can be seen that there is no significant difference between the regression results and the results of the original benchmark regression listed in Columns (1)–(2), indicating the excellent robustness of the benchmark regression results.

**Figure 1:**
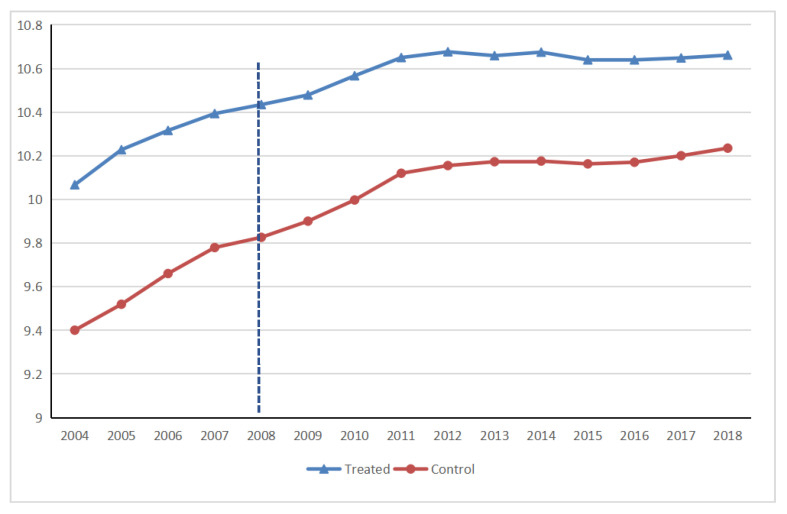
Common trend test results of air pollution control auditing.

The results of the common trend test are shown in Figure 1. As shown, the carbon emissions of treatment groups are larger than that of the control groups. The treatment groups and the control groups have consistent developing trends before 2008, and the gap between the two groups gradually narrows after 2008, indicating that the regression results are robust to a certain extent.

The propensity score matching is listed in Columns (5)–(6) of Table 6. As shown, the effect of air pollution auditing on carbon emissions is negative and significant, and that on carbon emission intensity is still not significant. It again proves the robustness of the initial benchmark regression results.

**Figure 2:**
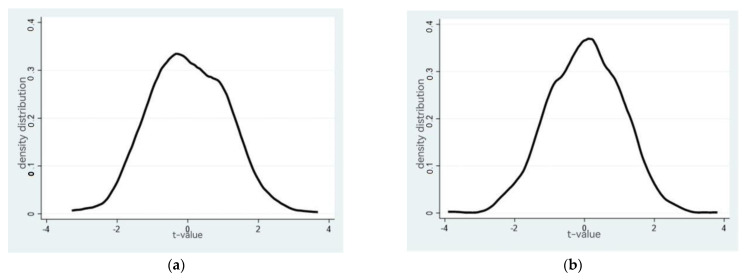
The density distributions of the coefficients of audit related to (**a**) carbon emissions; (**b**) carbon emission intensity obtained by the placebo test.

The density distributions of the coefficients of air pollution control auditing obtained by the placebo test are plotted in Figure 2. It is obvious that the coefficients estimated based on the randomly selected placebo group are also distributed mainly around the zero value, suggesting a slight influence from the missing variables on the regression results. Most of the critical variables were controlled by the original benchmark regression results.

## 5. Conclusions, Discussion, and Policy Implications

### 5.1. Conclusions

Based on 30 sets provincial panel data in China from 2004–2018, this paper explores the influence of air pollution control auditing on carbon emissions from the perspective of supervision. The study shows that (1) with full consideration of robustness and endogeneity, air pollution control auditing shows a significant effect on the reduction of carbon emissions. Specifically, the top-down audit is more effective than the bottom-up audit on carbon emissions. This may be due chiefly to the fact that the enthusiasm and execution efficiency of local audit institutions are greater than those of National Audit Office. (2) Air pollution control auditing has no significant influence on carbon emission intensity. Some provinces that control their economic growth rates to reduce energy consumption and sacrifice the economy to reduce carbon emissions may account for this situation. (3) The transmission mechanism suggests that air pollution control auditing urges local governments to perform a simple and direct method, such as reducing output and controlling shutdown, rather than promoting technological progress and green transformation of enterprises, thus reducing carbon emissions.

### 5.2. Discussion

The various local audit institutions at the provincial, municipal, and county levels, together with the National Audit Office, form a structured multi-tier auditing system with wide coverage. This paper focus on the influence of provincial-level air pollution control auditing and carbon emissions in China, without an in-depth discussion of China’s cities, which may result in some deviations. Exploring the impact of air pollution control auditing on carbon emissions based on the panel data of Chinese cities is a direction of future efforts.

Environmental auditing has a strong spatial spillover effect [67], which leads to the spatial correlation and dependence of regional carbon emissions. Although there have been many studies on the spatial spillover of carbon emissions [68,69], few studies focus on that from the perspective of air pollution control auditing. Therefore, future research should incorporate the air pollution control auditing into the spillover analysis framework and analyze the relationship between air pollution control auditing and the spatial spillover of carbon emissions.

This paper does not consider the regional heterogeneity of air pollution control auditing. In fact, the relatively developed regions may have been more willing to invest enough financial, material, and human resources to deal with local environmental pollution problems. In addition, auditors have relatively high enthusiasm, which is more conductive to promoting local carbon emission reduction. Therefore, it is expected to investigate the effect of the regional heterogeneity of air pollution control auditing on carbon emissions.

### 5.3. Policy Implications

According to the above findings, we make the following recommendations to help government and audit institutions to better promote carbon emission reduction effects and thus contribute to sustainable development.

Firstly, the audit institutions should strengthen the intensity and power of air pollution control auditing. As carbon emission reduction is a long-term, complex, and diversified task, audit institutions should deeply study the background, objective and requirements of the proposed policies to reduce carbon emissions. At the same time, audit institutions can enhance the combination of air pollution control auditing and other professional audits, innovating the auditing methods of air pollution control, and further improve the effectiveness of air pollution control auditing on carbon emission reduction.

Secondly, the coordination mechanism of audit institutions at different levels through system construction should be improved. The National Audit Office can play the leading role of national policies, and the local audit institutions promote the continuous improvement of the policy system based on the practical experience. Meanwhile, the local audit institutions need to recognize the importance of auditing for climate change and actively supervise the implementation of carbon emission reduction.

Finally, the government should explore more possible mechanisms to effectively control carbon emissions. It is not advisable to reduce carbon emissions at the expense of the national economy; the government can increase investment in air pollution control, providing external incentives, such as financial subsidies for pollution control, technological innovation, and green transform of enterprises, to promote enterprises to transform to the direction of low pollution and low energy consumption.

## Figures and Tables

**Table 1:** Carbon emission coefficients of various energy sources.

Energy Type	Conversion Coefficient of Standard Coal	Calorific Value of Standard Coal (kJ/kg)	Carbon Content per Unit Calorific Value (t-C/TJ)	Carbon Oxidation Rate	Carbon Emission Coefficient
Raw coal	0.7143	29,307	26.37	0.94	1.9027
Coke	0.9714	29,307	29.50	0.93	2.8638
Crude oil	1.4286	29,307	20.10	0.98	3.0240
Gasoline	1.4714	29,307	18.90	0.98	2.9286
Kerosene	1.4714	29,307	19.60	0.98	3.0371
Diesel oil	1.4571	29,307	20.20	0.98	3.0996
Power	0.1229	29,307	26.37	0.94	0.3274
Fuel oil	1.4286	29,307	21.10	0.98	3.1744
Natural gas	1.3300	29,307	15.30	0.99	2.1648
Liquefied petroleum gas	1.7143	29,307	17.2	0.98	3.1052

**Table 2:** Definitions of variables.

Variable	Index Selection	Symbol	Definition
Dependent variables	Carbon emission	Ln_Carbon	Logarithm of carbon emission
	Carbon emission intensity	Intensity	Carbon emission/GDP
Independent variables	Air pollution control auditing	AA	Whether the air pollution control auditing is implemented by the National Audit Office or local audit institutions
	Top-down audit	Cen.AA	Whether the air pollution control auditing is implemented by the National Audit Office
	Bottom-up audit	Loc.AA	Whether the air pollution control auditing is implemented by the local audit institutions
Mediating variables	Industrial added value	IVA	Logarithm of industrial value-added
	Industrial waste gas treatment capacity	faci	Logarithm of industrial waste gas treatment capacity
	Industrial pollution control waste gas project	pro	Number of waste gas projects for industrial pollution control
	Operating cost of industrial waste gas treatment facilities	cost	Logarithm of operation cost of industrial waste treatment facilities
Control variables	Economic development level	pgdp	GDP/population
	Industrial structure	Ind	The added value of the second industry/GDP
	Energy-resource structure	Energy	Coal consumption/energy consumption
	Technical level	Tech	GDP/energy consumption
	Urbanization level	Urban	Urban population/total population
	Environmental regulation	Regulation	Investment in industrial pollution source control/GDP

**Table 3:** Descriptive statistics of variables.

Variable	Sample	Mean	Med	Std. Dev.	Min	Max
ln_Carbon	450	10.20	10.20	0.805	7.129	11.91
Intensity	450	3.286	2.592	2.418	0.250	14.77
AA	450	0.333	0	0.472	0	1
Cen.AA	450	0.218	0	0.413	0	1
Loc.AA	450	0.162	0	0.369	0	1
pgdp	450	3.670	3.221	2.460	0.424	15.10
Urban	450	0.532	0.516	0.143	0.263	0.896
Ind	450	0.435	0.447	0.0812	0.165	0.620
Energy	450	0.951	0.890	0.389	0.0380	2.423
Tech	450	1.195	1.031	0.685	0.224	4.554
Regulation	450	0.00182	0.001	0.00165	6.4 × 10^−5^	0.0110
IVA	450	8.333	8.477	1.100	4.785	10.54
faci	450	10.40	10.36	1.230	6.937	14.87
pro	450	125.1	83	108.3	1	651
cost	450	12.39	12.52	1.152	8.006	14.96

**Table 4:** Benchmark regression results of the air pollution control auditing to reduce carbon emissions.

	(1)	(2)	(3)	(4)	(5)	(6)
Variable	Ln_Carbon	Intensity	Ln_Carbon	Intensity	Ln_Carbon	Intensity
AA	−0.021 **	0.021				
	(−2.18)	(0.20)				
Cen.AA			0.006	0.107		
			(0.58)	(0.92)		
Loc.AA					−0.029 **	−0.040
					(−2.57)	(−0.61)
Control variables	YES	YES	YES	YES	YES	YES
Region	YES	YES	YES	YES	YES	YES
Year	YES	YES	YES	YES	YES	YES
R^2^	0.881	0.742	0.881	0.743	0.882	0.742
Sample size	450	450	450	450	450	450

Note: ** represent 5% level of statistical significance. Standard errors are reported in parentheses.

**Table 5:** Bootstrap estimation of mediation effects.

Dependent Variables	Independent Variables	Mediating Variables	Effect Category	Effect Size	95% Error Corrected Confidence Interval		
					Standard Error	Lower Bound	Upper Bound
Ln_Carbon	AA	IVA	Indirect effect	−0.0099	0.0040	−0.0168	−0.0025
			Direct effect	−0.0113	0.0111	−0.0319	0.0194
		faci	Indirect effect	−0.0003	0.0011	−0.0011	0.0005
			Direct effect	−0.0210	0.0129	−0.0450	0.0030
		pro	Indirect effect	−0.0009	0.0011	−0.0041	0.0007
			Direct effect	−0.0203	0.0123	−0.0460	0.0056
		cost	Indirect effect	0.0017	0.0037	−0.0052	0.0116
			Direct effect	−0.0229	0.0122	−0.0442	0.0086
	Loc.AA	IVA	Indirect effect	−0.0081	0.0039	−0.0146	−0.0027
			Direct effect	−0.0208	0.0109	−0.0466	−0.0040
		faci	Indirect effect	0.0001	0.0010	−0.0004	0.0031
			Direct effect	−0.0290	0.0123	−0.0486	−0.0001
		pro	Indirect effect	−0.0007	0.0009	−0.0045	0.0002
			Direct effect	−0.0281	0.0125	−0.0525	−0.0037
		cost	Indirect effect	0.0005	0.0043	−0.0069	0.0114
			Direct effect	−0.0294	0.0115	−0.0410	0.0140

**Table 6:** Regression results after the replacement of independent variable and propensity score matching.

	(1)	(2)	(3)	(4)	(5)	(6)
Variables	Ln_Carbon	Intensity	Ln_Carbon	Intensity	Ln_Carbon	Intensity
AA	−0.021 **	0.021			−0.022 **	0.001
	(−2.18)	(0.20)			(−2.25)	(0.01)
AAF			−0.008 **	−0.006		
			(−2.10)	(−0.17)		
Control variables	YES	YES	YES	YES	YES	YES
Region	YES	YES	YES	YES	YES	YES
Year	YES	YES	YES	YES	YES	YES
R^2^	0.881	0.742	0.882	0.742	0.877	0.743
Sample size	450	450	450	450	450	450

Note: ** represent 5% level of statistical significance. Standard errors are reported in parentheses.
